# Short Stature Homeobox-Containing Haploinsufficiency in Seven Siblings with Short Stature

**DOI:** 10.1155/2017/7287351

**Published:** 2017-08-30

**Authors:** Elizabeth S. Sandberg, Ali S. Calikoglu, Karen J. Loechner, Lydia L. Snyder

**Affiliations:** ^1^Division of Endocrinology, Department of Pediatrics, University of North Carolina at Chapel Hill, Chapel Hill, NC, USA; ^2^Division of Pediatric Endocrinology, Department of Pediatrics, Children's Healthcare of Atlanta, Atlanta, GA, USA; ^3^Division of Pediatric Endocrinology, Department of Pediatrics, Nemours Children's Health System, Jacksonville, FL, USA

## Abstract

Deficiency of the short stature homeobox-containing (SHOX) gene is a frequent cause of short stature in children (2–15%). Here, we report 7 siblings with SHOX deficiency due to a point mutation in the SHOX gene. Index case was a 3-year-old male who presented for evaluation of short stature. His past medical history and birth history were unremarkable. Family history was notable for multiple individuals with short stature. Physical exam revealed short stature, with height standard deviation score (SDS) of −2.98, as well as arm span 3 cm less than his height. His laboratory workup was noncontributory for common etiologies of short stature. Due to significant familial short stature and shortened arm span, SHOX gene analysis was performed and revealed patient is heterozygous for a novel SHOX gene mutation at nucleotide position c.582. This mutation is predicted to cause termination of the SHOX protein at codon 194, effectively causing haploinsufficiency. Six out of nine other siblings were later found to also be heterozygous for the same mutation. Growth hormone was initiated in all seven siblings upon diagnosis and they have demonstrated improved height SDS.

## 1. Background

Deficiency of the short stature homeobox-containing (SHOX) gene is a frequent cause of short stature in children [1]. Early studies indicated a gene or group of genes relating to short stature in the pseudoautosomal region (PAR) of the X and Y chromosomes [2]. In 1997, Rao et al. isolated the SHOX gene within a region in the pseudoautosomal region 1 (PAR1) that was deleted in multiple individuals with short stature, but not deleted in relatives with normal stature [3], at around the same time that Ellison et al. isolated a gene from the PAR region that was a candidate gene for the short stature of Turner syndrome [4]; both eventually would be identified as the SHOX gene. Ultimately, it was discovered that a dose-dependent relationship exists between an individual's height and the number of actives copies of the SHOX gene. Thus, SHOX haploinsufficiency is associated with short stature (as seen with Turner syndrome), and additional SHOX genes (as seen with sex-chromosome polyploidies such as Klinefelter syndrome, triple X syndrome, and XYY syndrome) are associated with tall stature [5]. SHOX deficiency is also the primary cause of short stature in most patients with Leri-Weill dyschondrosteosis. Loss of both SHOX alleles causes an extreme phenotype of osteodysplasia, which is also called Langer syndrome [1]. For children with idiopathic short stature, the prevalence of SHOX mutations is estimated to be within 2–15% [1].

The most common mutations involving the SHOX gene are deletions of various sizes which encompass either the SHOX gene itself or a regulatory enhancer region [1]. These deletions account for approximately two-thirds of all mutations, while point mutations are less common, making up approximately one-third of all mutations [6]. Here we present a family with short stature, of which 7 of 10 siblings were found to be heterozygous for a point mutation in the SHOX gene.

## 2. Case Presentation

### 2.1. Case 1

This Latino male is the third oldest child of a family originally from Mexico. He presented at the age of 3 years and 2 months for evaluation of short stature (Table 1).

He was born at full term, after an uncomplicated pregnancy and delivery, and his birth weight was near the 10th percentile. As an infant, his height initially was at the 10th percentile, then dropped below the 5th percentile at 22 months of life, and was far below the 3rd percentile by 33 months of life. He maintained his weight around the 10th percentile for the first 3 years of life. Family history is notable for maternal height of 143 cm (<3rd percentile) and paternal height of 156 cm (<3rd percentile), resulting in mid-parental target height of 156 cm. He also had five siblings with reported short stature and two siblings of normal stature.

On physical exam, his height was 85.4 cm (below 3rd percentile, height SDS −2.98), weight 11.8 kg (weight SDS −2.06). His arm span was 82.3 cm. He had no dysmorphic features. His testes were descended bilaterally, and he had a normal phallus.

Initial workup done for common etiologies for short stature revealed normal IGF-I, IGFBP-3, electrolytes, liver function tests, complete blood count, thyroid studies, sedimentation rate, and urinalysis. His bone age was congruent with his chronological age. No Madelung deformity was noted clinically or radiologically.

**Figure 1:**
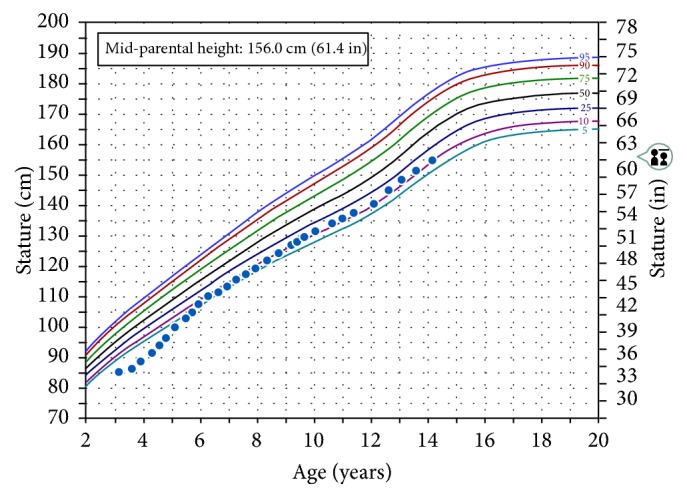
Height growth chart for Case 1.

Because of his severe short stature, family history, and short arm span, a SHOX gene analysis was performed, which revealed a p.C194X sequence variant at nucleotide position c.582 in the SHOX gene, changing a nucleotide from C to A (c.582C>A) in codon 194. This creates a termination codon, which is predicted to cause truncation of the SHOX protein at codon 194. He was heterozygous for this nonsense mutation. The patient was started on growth hormone (GH) at 39 *μ*g/kg/day, with resultant increased growth velocity (Figure 1). Height SDS improved to −1.06 after 9-year treatment (Table 1).

### 2.2. Cases 2–7

Six siblings of Case 1 subsequently presented for evaluation of short stature. The cases are listed in order of presentation to clinic, which occurred as they demonstrated decrease in growth velocity. Their clinical findings at presentation are summarized in Table 1.

**Figure 2:**
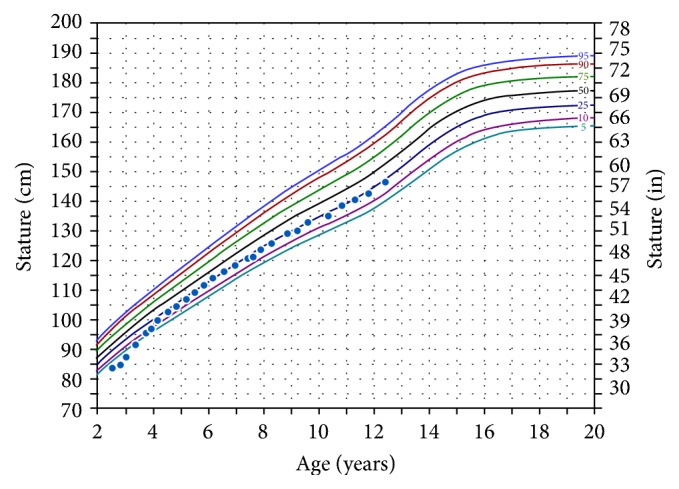
Height growth chart for Case 2.

All siblings were noted to have height SDS less than −2.0 at the time of presentation. None of the siblings were dysmorphic. Cases 2 and 3 were the only siblings to have any additional abnormal phenotypes diagnosed radiologically: a shortening of the ulna in Case 2, with short 4th and 5th metacarpals, and Madelung deformity of the radial epiphysis in Case 3. Bone ages were either normal or slightly delayed, and workup for other etiologies of short stature was negative in all individuals, as with Case 1. Due to the confirmed family history for SHOX mutation, the SHOX gene analysis was performed in all individuals, and all 6 siblings were found to be heterozygous for the same, novel mutation. Growth hormone treated was initiated in all cases (starting dose 36–45 *μ*g/kg/day), leading to improved height SDS for all cases (Table 1). A representative growth chart is provided in Figure 2.

**Figure 3:**
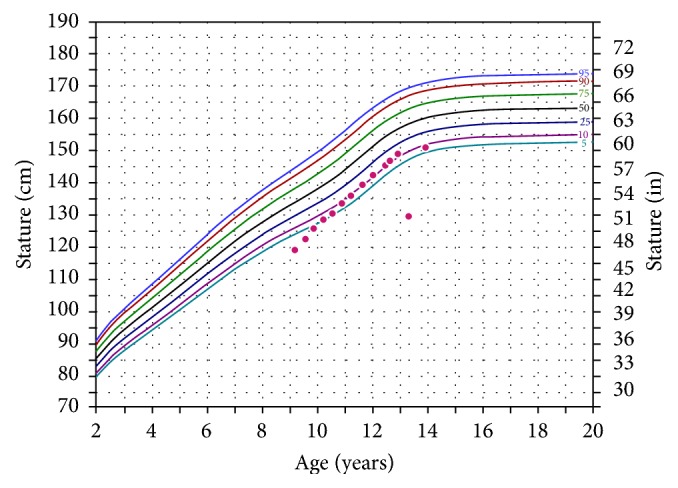
Height growth chart for Case 3.

Case 3 was the eldest child, and her GH treatment was discontinued at age 13 years and 4 months as she neared completion of linear growth after menarche (Figure 3). At that time, her height SDS was −1.42, improved from −2.48 at the initiation of GH treatment. Otherwise, GH treatment is ongoing in all cases.

## 3. Conclusions

This report describes a family with seven siblings with severe short stature at presentation. All are heterozygous for a point mutation of the short stature homeobox-containing (SHOX) gene that was included in SHOX database (http://grenada.lumc.nl/LOVD2/MR/home.php) but corresponding phenotype was not reported. The SHOX gene is located in the telomeric part of the pseudoautosomal region 1 (PAR1) region on the short arm of both sex chromosomes and does not undergo X inactivation [7]. Because of this, the SHOX gene is expressed on both X and Y chromosomes [1]. The SHOX gene encodes a transcription factor that appears to play a role in regulating chondrocyte differentiation and proliferation during early fetal life [7].

All cases had a mutation in exon 5, codon 194 (c.582C>A), that creates a termination codon. This results in a truncated protein and presumed haploinsufficiency. This truncated protein could potentially interfere with normal SHOX activity, but, at this time, the true mechanism is unknown. There are reports of mutations in the SHOX gene homeodomain affecting nuclear translocation [8]; however, given the location of the mutation in the 5th exon, we believe effective haploinsufficiency is the most likely explanation in this family. Phenotypically, this haploinsufficiency resulted in short stature, as is seen with other patients with SHOX deficiency.

SHOX deficiency, in addition to short stature, has a wildly varying phenotype, some features of which can act as a clue to the diagnosis. The Madelung deformity is a “spontaneous subluxation of the distal ulna forward” which results in the hand and forearm resembling a dinner fork [1]. Additional, less-specific signs include shortened 4th and 5th metacarpals, highly arched palate, increased carrying angle of the elbow, scoliosis, micrognathia, and muscular hypertrophy [1]. Of note, the absence of any of these signs would not exclude a diagnosis of SHOX haploinsufficiency. Surprisingly in this family, only 2 individuals showed additional phenotypic changes, other than short stature, seen in individuals with SHOX deficiency: Case 3 has short 4th and 5th metacarpals and a Madelung deformity, and Case 2 has shortening of the ulna. It should be noted, however, that Case 3 is the oldest sibling and the severity of skeletal deformities tends to worsen with puberty [7], which may explain these skeletal deformities in the oldest sibling.

The decision to obtain genetic testing in Case 1 was made based on the combination of his severe short stature and shortened arm span. His siblings were subsequently tested based on their known family history and significant short stature. When deciding when to pursue testing for SHOX deletion, there are a variety of clinical features to consider. Binder et al. [9] demonstrated that patients with SHOX deletion have shortened arm span for age, as well as one characteristic radiological sign of Leri-Weill-dyschondrosteosis, and concluded that these would be reasonable features to trigger initial SHOX gene testing in a patient with idiopathic short stature. Rappold et al. [5] developed a phenotype scoring system to aid this decision making, based on 8 dysmorphic features and anthropometric measurements including elevated sitting height/height ratio. Malaquias et al. [10] demonstrated that an elevated sitting height/height ratio alone is a useful tool for selecting short children for SHOX analysis. Due to medical record limitations, these additional measurements are unavailable in these 7 cases.

Using the rationale that girls with Turner syndrome, whose short stature is believed to be due, at least in part, to SHOX haploinsufficiency, have had good response to GH treatment [8], GH was later evaluated in children with isolated SHOX deficiency. Blum et al. [7] evaluated the efficacy of GH therapy in children with short stature and SHOX deficiency in a randomized, controlled trial. The GH treatment, dosed at 50 *μ*g/kg, resulted in significant increase in first- and second-year height velocities and in height SDS. Blum et al. [11] later found that GH treatment produces similar height gains for patients with SHOX deficiency when compared to girls with Turner syndrome. SHOX deficiency became an FDA approved indication for GH therapy in 2006 [9]. In this family, GH treatment was initiated upon diagnosis of SHOX deficiency related short stature, given the literature on the benefits on height gains in this patient population [11–13].

Consistent with previous observations, our patients all have a trend of improvement in their height SDS. In this family, there are a wide variety of ages at GH treatment onset as well as varied duration of GH treatment; however, we see that the two cases with the largest improvement in their SDS scores (Cases 1 and 2) also had the two longest durations of GH therapy. Based on the literature of girls with Turner syndrome, this is not surprising, as we would expect that the longer duration of treatment with GH would tend to lead to better improvement in height SDS [8]. This emphasizes the importance of early diagnosis of SHOX deficiency and early start of GH treatment before puberty triggers epiphyseal closure. In fact, Scalco et al. [14] demonstrated benefit of combining GH with GnRH therapy in children with SHOX defects who had just started puberty, to limit loss of growth potential triggered by puberty, and allow longer treatment duration.

Although there is known benefit of early initiation of GH therapy in older children, there is no precedent to direct the timing of GH initiation in a younger patient with known SHOX deficiency. Davenport et al. [15] showed that early GH treatment can correct growth failure and normalize height in infants and toddlers with Turner syndrome without significant adverse effect. Also, De Schepper et al. [16] studied the use of GH in very young children (<30 months) with failed catch-up growth after being born small for gestational age and found no significant negative impact mental or motor development in the very young children treated with GH, compared to the controls. Given these observations, three of the siblings (Cases 5, 6, and 7) were started on GH even before the age of 2, in an effort to maximize benefit of GH therapy on height.

In this family, the parents were never tested for SHOX deficiency. Presumably, one of the parents is heterozygous for this novel mutation and likely the proband for this mutation. Assuming the second parent is unaffected, a 50% inheritance rate would be anticipated, though in this family the inheritance rate was higher, at 70%, creating a unique opportunity to study 7 siblings from the same family.

In summary, SHOX deficiency should be considered in the differential diagnosis of children with severe short stature even in the absence of typical phenotypic features. A short parent, as well as other siblings with short stature, may increase the possibility of SHOX deficiency. Unfortunately, the diagnosis can be done only by genetic testing, which is costly and may not be available in many countries, and, as a result, testing should be reserved for children for whom there is a moderately high clinical suspicion for SHOX deficiency. GH treatment improves height with more favorable outcome when it is used for longer duration prior to epiphyseal fusion.

## Figures and Tables

**Table 1:** Results of growth hormone (GH) treatment in seven siblings with short stature due to SHOX haploinsufficiency.

Case	Sex	Age at onset of GH (years)	Bone age at onset of GH (years)	Duration of GH treatment (years)	Height SDS at initiation of GH	Height SDS in June 2014	Change in Height SDS
1	M	3.8	3.5	9.3	−3.1	−1.06	+2.04
2	M	2.9	1.5	8.5	−2.71	−0.77	+1.94
3	F	9.3	8.8	4.5	−2.48	−1.42	+1.06
4	M	10.6	10	4.3	−2.42	−1.83	+0.59
5	F	1.8	2	4.3	−2.57	−2.23	+0.34
6	F	1.5	1.3	3.5	−3.00	−2.52	+0.48
7	M	1.8	2	2.1	−2.26	−1.41	+0.85

## Abbreviations

SHOX: Short stature homeobox-containing
GH: Growth hormone.
